# Polarization of Immune Cells in the Pathologic Response to Inhaled Particulates

**DOI:** 10.3389/fimmu.2020.01060

**Published:** 2020-06-17

**Authors:** Qiang Ma

**Affiliations:** Receptor Biology Laboratory, Toxicology and Molecular Biology Branch, Health Effects Laboratory Division, National Institute for Occupational Safety and Health, Centers for Disease Control and Prevention, Morgantown, WV, United States

**Keywords:** polarization of immune cells, particle, nanoparticle, pulmonary inflammation and fibrosis, T helper, Treg, ILC, MDSC

## Abstract

Polarization of immune cells is commonly observed in host responses associated with microbial immunity, inflammation, tumorigenesis, and tissue repair and fibrosis. In this process, immune cells adopt distinct programs and perform specialized functions in response to specific signals. Accumulating evidence indicates that inhalation of micro- and nano-sized particulates activates barrier immune programs in the lung in a time- and context-dependent manner, including type 1 and type 2 inflammation, and T helper (Th) 17 cell, regulatory T cell (Treg), innate lymphoid cell (ILC), and myeloid-derived suppressor cell (MDSC) responses, which highlight the polarization of several major immune cell types. These responses facilitate the pulmonary clearance and repair under physiological conditions. When exposure persists and overwhelms the clearance capacity, they foster the chronic progression of inflammation and development of progressive disease conditions, such as fibrosis and cancer. The pulmonary response to insoluble particulates thus represents a distinctive disease process wherein non-infectious, persistent exposures stimulate the polarization of immune cells to orchestrate dynamic inflammatory and immune reactions, leading to pulmonary and pleural chronic inflammation, fibrosis, and malignancy. Despite large variations in particles and their associated disease outcomes, the early response to inhaled particles often follows a common path. The initial reactions entail a barrier immune response dominated by type 1 inflammation that features active phagocytosis by M1 macrophages and recruitment of neutrophils, both of which are fueled by Th1 and proinflammatory cytokines. Acute inflammation is immediately followed by resolution and tissue repair mediated through specialized pro-resolving mediators (SPMs) and type 2 cytokines and cells including M2 macrophages and Th2 lymphocytes. As many particles and fibers cannot be digested by phagocytes, resolution is often extended and incomplete, and type 2 inflammation becomes heightened, which promotes interstitial fibrosis, granuloma formation, and tumorigenesis. Recent studies also reveal the involvement of Th17-, Treg-, ILC-, and MDSC-mediated responses in the pathogenesis caused by inhaled particulates. This review synopsizes the progress in understanding the interplay between inhaled particles and the pulmonary immune functions in disease pathogenesis, with focus on particle-induced polarization of immune cells and its role in the development of chronic inflammation, fibrosis, and cancer in the lung.

## Introduction: the Immune Connection of Inhaled Particles

Humans maintain the systemic oxygen and carbon dioxide levels through respiration. During respiration, human lungs inhale and exhale ~7,200–11,520 liters of air each day to exchange the gases with the blood across the surface of about 300 million alveoli (1, 2). At the same time, respiration exposes the lung to a myriad of potentially harmful airborne agents, including microbes, allergens, and inorganic particulates, from inhaled air on a daily basis (3–6). The pulmonary immune system must recognize these microbial and non-microbial threats and react swiftly to destroy and clear them from the lung in order to keep the airway and alveolar space clean and healthful, which is essential for the proper ventilation, gas exchange, and host defense in the lung. For this purpose, the lung has evolved with delicate and unique barrier immune mechanisms that enable it to defend against inhaled pathogens and non-infectious insults effectively. Loss or exacerbation of the lung barrier immunity is known to have a major impact on the development of lung disease caused by microbial and some non-microbial exposures (5–9).

The respirable inorganic particulates consist of a large group of particles, fibers, dusts, and engineered nanomaterials (ENMs). These micro- and nano-sized, insoluble particulates represent a unique threat to pulmonary structure and function. Humans are constantly exposed to these particulates through polluted air, automobile exhaust, industrial and occupational exposures, consumer products, and natural and man-made disastrous events. Because of their small size and airborne propensity, these particles and fibers can reach small airways and alveoli upon inhalation, with some penetrating into the interstitial space, pleural cavity, and blood circulation, causing a range of disease conditions, such as inflammation, fibrosis, cancer, and autoimmune dysfunction, in the lung and pleura, and some in extrapulmonary organs (4, 10–12).

A link between inhaled particulates and respiratory illness was noted as early as the 15th and 16th century. Nonetheless, it was not until the 19th and early 20th centuries that inhaled particles and dusts were established as the direct cause of several deadly lung diseases, including silicosis and accompanying tuberculosis, black lung disease and associated massive progressive fibrosis (MPF), and particle-associated lung cancer and mesothelioma (10, 13). Crystalline silica, asbestos, and coal dust were the three major particulate hazards that exerted a major toll of death and morbidity in many western countries during their rapid industrialization. Although exposure to these prototypical industrial dusts has been greatly reduced for decades, particle-induced lung disease continues to be a substantial health threat to workers and populations at large in many developing countries and to some professions in developed countries worldwide (14–17). Moreover, the rapid development of ENMs in the past two decades has raised new concerns, as certain nanomaterials, such as carbon nanotubes (CNTs), were found to cause pulmonary inflammation, fibrosis, and cancer in exposed animals, with some having potencies larger than those of silica and asbestos (4, 11, 18). On the other hand, our understanding of how inhaled particles cause progressive lung disease remains largely limited at the level of anatomical pathology despite decades of intensive research. In part, this lack of mechanistic insights into particle toxicity is responsible for the nonexistence of effective therapy against lung disease caused by particulates. As such, these diseases are frequently progressive and refractory to therapy, giving rise to high rates of mortality and disability. For these reasons, there is substantial, renewed interest in understanding the fundamental aspects of the interaction between inhaled particles and human lungs and how it leads to diverse diseases.

Establishing the connection between pulmonary immunity and disease pathogenesis by inhaled particulates is not intuitive, as inorganic particulates do not possess antigenic structures like microbial proteins and allergens. Accordingly, the interaction between the immune system and inhaled particulates in the lung has long being considered as one that is passive and simple. In this framework, inhaled particles are cleared from the lung rapidly and passively by innate immune mechanisms, which consist of phagocytosis by sentinel alveolar macrophages (AMs), mucociliary escalator transport by airway epithelia, and drainage into the local lymph nodes. In the process, engulfing phagocytes can be poisoned by particulates as inorganic particles cannot be digested by the phagocytes. While the importance of these particle-innate immune interactions in the pulmonary clearance of inhaled particles is well recognized, little is known about how the interaction between inhaled particles and the pulmonary immune system contributes to disease development. In this regard, several lines of recent evidence and progress provided new insights into the role of pulmonary immunity in the pathogenesis of lung disease caused by inhaled particles.

A number of epidemiological studies have identified an association between exposure to silica and increased incidence of autoimmune disorders, including systemic lupus erythematosus, systemic sclerosis, and rheumatoid arthritis, in exposed workers in comparison to the general population (12, 19–23). Exposure to silica or asbestos also increased the production of autoantibodies and caused hypergammaglobulinemia in individuals that did not have a diagnosed autoimmune disease (24). The mechanism by which inhaled particles cause autoimmune reactions and disease is not well understood. Experimental evidence suggested a role of particle-induced cell death, inflammation, and release of tissue antigens, which may account for the production of autoantibodies, but does not readily explain how self-tolerance is breached and autoimmune disease is induced from particle exposure (12, 25). In the meantime, a large body of evidence has been obtained to demonstrate that pulmonary exposure to silica, asbestos, and nanomaterials stimulates polarized immune and inflammatory responses in exposure-, time-, and context-dependent manners. These responses resemble aspects of the pulmonary immunity against inhaled pathogens and allergens, including type 1 and type 2 immunity/inflammation, and T helper (Th) 17 cell, regulatory T cell (Treg), innate lymphoid cell (ILC), and myeloid-derived suppressor cell (MDSC), responses. Central to these polarized immune reactions is the polarization of some major immune cells, such as subtypes of CD4+ Th cells, Tregs, ILCs, classically or alternatively activated (M1 or M2) macrophages, and MDSCs. Under physiological conditions, these responses facilitate the clearance of inhaled particles, repair of damaged lung tissue, and return of pulmonary homeostasis. When exposure persists and overwhelms the clearance capacity of the lung, these immune reactions foster the chronic progression of inflammation and development of progressive disease conditions, such as fibrosis and cancer. In this regard, the pulmonary response to insoluble particulates represents a distinctive disease process in which non-infectious, persistent exposures stimulate the polarization of immune cells to orchestrate dynamic inflammatory and immune responses, leading to pulmonary and pleural chronic inflammation, fibrosis, and malignancy, as well as systemic autoimmune dysfunction. Therefore, understanding the interplay between inhaled particulates and the pulmonary immune functions is key to the mechanistic study, risk assessment, and drug targeting against particle-induced chronic and progressive diseases.

This review summarizes recent advances that highlight immune mechanisms in the development of pulmonary inflammation, fibrosis, and cancer caused by exposure to inhaled micro and nano particulates. The review focuses on induced polarization of immune cells and their responses, which is believed to play a major role in disease pathogenesis by inhaled particles. This body of knowledge would facilitate the safety evaluation, biomarker identification, and therapeutic targeting against inhaled particulates. For discussion on the autoimmune effects of inhaled particles, readers are referred to several excellent, dedicated reviews (12, 19, 21, 22, 24, 25).

## Polarization of Immune Cells in Pulmonary Barrier Immunity and Disease

Much of our understanding of the pulmonary barrier immunity derives from studies on immunity against pathogens and allergens (5, 6, 26–28). Therefore, it is useful to briefly review some concepts and mechanisms involved in pulmonary microbial immunity and allergy that are pertinent to particle clearance and pathogenesis.

Inhaled pathogens and allergens stimulate immune reactions in the lung through complex and delicate interplays between resident and infiltrating, innate and adaptive, immune cells. These interactions lead to the production of secreted mediators, including cytokines, chemokines, growth factors, lipid mediators, and reactive chemicals. Together, these cellular and molecular mediators shape the tissue response to exposed pathogens and allergens, including inflammation, pulmonary clearance, and tissue repair. Epithelial cells, dendritic cells (DCs), and AMs are among the first line of cells to contact exposed microbes (27, 28). These sentinel cells recognize microbial antigens and pathogen-associated molecular patterns (PAMPs) through cell surface and intracellular receptors, such as Toll-like receptors (TLRs) and pattern recognition receptors (PRRs). The PRR-activated inflammasomes integrate a wide range of stimulating signals to boost proinflammatory responses by promoting the maturation and secretion of interleukin (IL)-1β and IL-18. DCs present antigenic signals to T cells and activate adaptive immunity, such as B cell activation and immunoglobulin (Ig) class-switching. Innate immune cells initiate downstream inflammatory and immune responses by releasing danger-associated molecular patterns (DAMPs) and proinflammatory mediators. These signals coordinate the recruitment of inflammatory and immune cells to the site of infection. Neutrophils infiltrate immediately and mediate short-term immune-pathogen interactions, whereas monocytes, macrophages, and lymphocytes predominate later responses and promote the resolution of inflammation and wound healing in addition to pathogen clearance. When this pulmonary barrier immunity is compromised, the lung becomes a port of entry for inhaled pathogens to invade and proliferate, causing different types of lung infections and allergic reactions, exemplified by bacterial and viral pneumonia, tuberculosis, and asthma.

**Figure 1:**
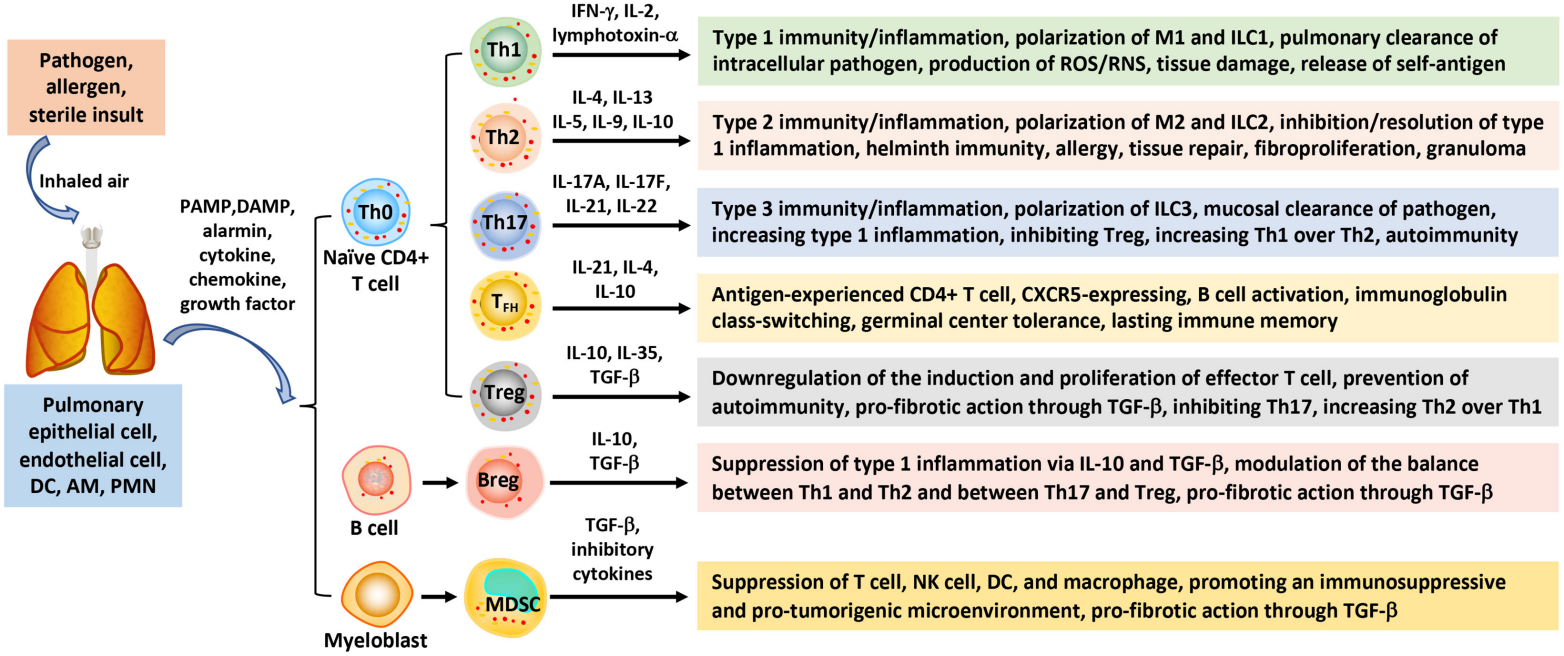
Polarization of immune cells and responses in pulmonary barrier immunity. Inhaled pathogens, allergens, and sterile insults stimulate barrier immune and inflammatory responses in the airways and lung parenchyma through polarized innate and adaptive immune cells. Interaction between the inhaled instigators and sentinel cells (epithelial and endothelial cells, DCs, and macrophages), as well as infiltrating PMNs, generates PAMPs, DAMPs, alarmins, and pro-inflammatory mediators that trigger the activation and polarization of immune cells. Naïve CD4+ T lymphocytes (Th0) can differentiate into Th1, Th2, Th17 subpopulations that mediate type 1, type 2, and type 3 immunity/inflammation, respectively. CD4+ Th0 cells can also polarize to T_FH_ that mediate B cell activation and class-switching, and Treg that down-regulate effector T cell effects. B lymphocytes may polarize to Breg to suppress type 1 inflammation, whereas MDSC cells derived from perturbed myelopoiesis exhibit immunosuppressive, pro-tumorigenic, and pro-fibrotic activities. AM, alveolar macrophage; Breg, regulatory B; DAMP, danger-associated molecular pattern; DC, dendritic cell; IFN, interferon; IL, interleukin; ILC, innate lymphoid cell; MDSC, myeloid-derived suppressor cell; PAMP, pathogen-associated molecular pattern; PMN, polymorphonuclear leukocyte; T_FH_, follicular helper T; TGF, transforming growth factor; Th, T helper; Treg, regulatory T.

In both normal and pathologic pulmonary responses, the respiratory immune system is tailored to respond to different classes of pathogens and allergens optimally through disparate immune reactions commonly known as type 1, type 2, and type 3 immunity (Figure 1) (29, 30). In many chronic disease conditions and during non-infectious exposures, inflammation often predominates the immune responses. In these cases, the immune responses are sometimes called type 1, type 2, and type 3 inflammation, respectively.

Type 1 immunity is characterized by Th1 cells and ILC group 1 cells, which secrete interferon (IFN) γ, IL-2, and lymphotoxin-α. Type 1 responses protect against intracellular microbes through activated mononuclear phagocytes, i.e., M1 macrophages, and an array of proinflammatory cytokines, eicosanoids, and reactive oxygen species (ROS) and reactive nitrogen species (RNS), to stimulate acute inflammation and bacterial killing. Heightened type 1 responses cause excessive damage to lung tissue and contribute to disease pathogenesis, including releasing self-antigens that induce autoimmune reactions.

Type 2 immunity consists of Th2 cells, ILC2s, and M2 macrophages, which secrete type 2 cytokines, such as IL-4, IL-5, IL-9, IL-10, and IL-13. These cytokines recruit and activate type 2 effector cells, including eosinophils, basophils, mast cells, and myofibroblasts. Some Th2 cells migrate to lymph node follicles and promote IgE class switch and B cell activation and, hence, are called follicular helper T cells (T_FH_s). Type 2 responses protect against helminth infection, venoms, and allergens under physiological conditions but, when dysregulated, lead to atopic responses, such as asthma and anaphylaxis. Recent studies reveal that ILC2s can be activated in response to a wide range of stimuli. Moreover, activated ILC2s secrete copious amounts of type 2 cytokines prior to Th2 activation (31). These findings suggest a mechanism by which type 2 responses can be initiated in the absence of apparent antigenic stimulation. Functionally, type 2 immunity has been traditionally associated with allergic responses, host defense against helminth infection, and tissue repair. Recent findings suggest a contemporary view of type 2 functions in which type 2 responses seemingly play a more general role in defense against noxious environmental stimuli besides mediating host immunosurveillance at barrier sites. In this context, type 2 reactions help eliminate, restrict, and neutralize noxious environmental substances and triggers, such as allergens, as well as repair tissue damage and minimize inflammation at surface tissue. Key to the function of type 2 responses in tissue regeneration, wound healing, and suppression of type 1 inflammation is the production of transforming growth factor (TGF) β by type 2 cells, such as M2 macrophages. Additionally, ILC2s, eosinophils, and type 2 cytokines are vital regulators of adipose precursor number and fate and the overall adipose tissue homeostasis (32). This innate type 2 immune metabolic circuit regulates energy metabolism and, thereby, controls insulin sensitivity and lean physiology (33). Therefore, the current view of the biology and scope of type 2 immunity has expanded considerably beyond the traditionally recognized type 2 responses (34).

Type 1 and type 2 responses are mutually suppressive to each other in many cases and hence are dichotomous, which helps orchestrate the temporal development of host responses to infection and inflammatory instigators. Apart from this widely accepted paradigm of type 1 and type 2 immunity, a type 3 immune response has been implicated in pulmonary immunity. Type 3 immunity is characterized by Th17 cells and ILC3s, and the production of IL-17 and IL-22 cytokines. Type 3 immune responses protect against extracellular bacteria and fungi through mononuclear phagocytes and neutrophils at mucous membrane epithelia (30).

Central to these three types of immunity/inflammation is the polarization of several major immune cells, including T lymphocytes, macrophages, and ILCs, induced by microbial signals, allergens, sterile insults, and microenvironmental cues from damaged tissue (Figure 1). During polarization, innate and adaptive immune cells adopt distinct programs to obtain unique properties and perform specialized functions in associated immune responses (30). Naïve CD4+ T, i.e., Th0, cells can differentiate into Th1, Th2, or Th17 cells in response to specific stimulating signals. Polarized Th cells play critical roles in the initiation, amplification, and resolution or progression of type 1, typ2, or type 3 immunity, accordingly. Similarly, ILCs can polarize into ILC1, ILC2, and ILC3 subpopulations to regulate associated immune responses.

Polarization of immune effector cells is best exemplified by the induced differentiation of macrophages into M1 and M2 macrophages during type 1 and type 2 immune responses, respectively (35–38). M2 cells can be further separated into distinctive 2a, 2b, 2c, and 2d subgroups according to their activating signals, secreted cytokines, and activities. IL-4 and IL-13 are major inducers of M2a polarization. M2a cells regulate tissue repair and the internalization of proinflammatory molecules by upregulating the expression of arginase-1 (ARG1), mannose receptor C-type 1 (MRC1, CD206), major histocompatibility complex (MHC) II, and IL-10 and TGF-β. M2b cells are activated by immune complexes or lipopolysaccharides (LPS) and produce IL-1, IL-6, IL-10, and TNF-α to activate Th2 cells and anti-inflammatory activities. M2c cells are activated in response to IL-10, TGF-β and glucocorticoids. M2cs produce IL-10 and TGF-β to suppress inflammatory responses. M2ds, which are activated by IL-6 and adenosine, are associated with tumor microenvironment and, hence, are named tumor-associated macrophages (TAMs). Polarization of TAMs is regulated by cell signaling molecules, such as IL-4 and IFN-γ, as well as extracellular metabolites, such as lactate, in the tumor microenvironment (39). Of note, polarization of macrophages exhibits considerable plasticity with regard to their cell source, inducing signal, mechanism of differentiation, and interconversion between subtypes (35–38).

In addition to type 1, type 2, and type 3 responses, Treg lymphocytes can be enriched to regulate immune responses by controlling the polarization of Th1, Th2, and Th17 effector T (T eff) cells and hence the balance among these immune responses. MDSCs are differentiated from bone marrow precursors as a result of perturbed myelopoiesis caused by pathogens and malignancy. MDSCs are characterized by their strong inhibitory activities toward T eff cells. Consistent with these major functions of polarized immune cells in the initiation and regulation of different types of immunity, a large body of evidence supports critical roles of immune cell polarization in microbial immunity and the pathogenesis of a wide range of diseases, including infection, autoimmunity, chronic inflammation, fibrosis, malignancy, atherosclerosis, neurodegeneration, and pertinent to this review, wound healing and fibrosis. For more detailed discussions on pulmonary microbial immunity and allergy, readers are referred to specialized reviews published elsewhere (29, 30, 34, 40, 41).

## Inflammation as a Common Response to Inhaled Particles

Inhaled particulates can cause a diverse range of disease phenotypes in humans and animal models. Among them, chronic inflammation, fibrosis, and cancer predominate and are of particular concern, as these pathologic conditions frequently adopt a progressive course to lead to severe outcomes (18). The pathogenesis of these chronic pathologic outcomes is complex and would differ from one another. Nevertheless, there clearly exist certain common features in the pathological effects caused by different types of particulates. These are exemplified by the presence of prominent granulomatous inflammation and interstitial fibrosis in the parenchyma of lungs exposed to inhaled particles and nanoparticles, and mesothelioma and pleural plague caused by inhaled asbestos fibers and carbon nanotubes. These common pathologic phenotypes suggest shared mechanisms in the pathogenesis of particulate-induced illnesses, which are seemingly attributable to the interactions between inhaled particles and the pulmonary immune system (12, 42, 43).

Pulmonary inflammation is a common and often the most prominent early response to inhaled particulates, provided an appropriate dose is received. Highly toxic particulates, exemplified by crystalline silica, crocidolite asbestos, and some CNTs, such as the Mitsui multi-walled CNTs (MWCNTs), i.e., XNRI MWNT-7, that have a fiber-like shape with high rigidity, stimulate apparent acute inflammatory responses in the airway and parenchyma at a low dose (44–46). On the other hand, poorly soluble particles of low toxicity (PSLTs), such as coal dust and amorphous carbon black, require substantially higher doses than those of silica and asbestos—often in one or more orders of magnitude—to induce evident inflammation. Mechanistically, acute inflammation is mediated mainly through the actions of innate immune cells, blood vessels, and molecular mediators. The acute response is characterized by increased movement of the plasma and blood leukocytes, mostly neutrophils, but also basophils, eosinophils, monocytic phagocytes, mast cells, and lymphocytes, which migrate across permeabilized micro blood vessels to tissues where particles deposit and injuries occur. A variety of proinflammatory cytokines, chemokines, growth factors, eicosanoids, and reactive chemicals are produced from activated immune cells and injured epithelial, endothelial, and fibroblastic cells. Proinflammatory mediators are secreted into the interstitial, alveolar, and pleural spaces to regulate the cellular, vascular, and matrix reactions. Some inflammatory mediators, such as ROS and RNS like nitric oxide (NO), are potently cytotoxic and can cause substantial damage to lung tissue if acute inflammation is heightened or prolonged. Therefore, acute inflammation is generally followed by resolution events, which avoid excessive tissue damage and promote recovery of tissue homeostasis. A combination of mediators and events mediate the resolution of inflammation, including specialized pro-resolving mediators (SPMs) and cytokines IL-10 and TGF-β. SPMs inhibit proinflammatory activities, such as the recruitment of neutrophils, and, at the same time, promote active pro-resolving activities, including efferocytosis of dead neutrophils and injured epithelia by macrophages and repair of damaged tissue through activated fibroblasts and myofibroblasts (47).

The role of inflammation in the development of particle-induced chronic outcomes has long been a subject of debate. It has been generally believed that the continued presence of particles and inflammation in lung tissue stimulates fibroproliferation and matrix production and remodeling, which promote fibrosis and tumorigenesis. This view of a causative relationship between inflammation and pathogenesis of fibrosis and malignancy is perhaps overly simple, because anti-inflammatory therapy has not proven to be beneficial in treating these diseases clinically. On the other hand, recent studies have provided new, significant, mechanistic insights into the inflammatory response to inhaled particles. These include a time-dependent development of type 1 and type 2 inflammation, the activation and function of several lymphocytic and myeloid immune cell-mediated immune responses, and a better understanding of the molecular basis for inflammation resolution and chronic progression. Moreover, these reactions have been shown to play critical roles in the development of fibrosis, cancer, and autoimmune dysfunction (18, 25, 42, 43). From this prospect, our understanding of the pulmonary inflammatory responses to inhaled particles has changed considerably in both their underlying immune mechanisms and relevance to disease pathogenesis, which are discussed in more details in the following sections.

## Type 1 Inflammation in Pulmonary Particle Clearance and Injury

**Figure 2:**
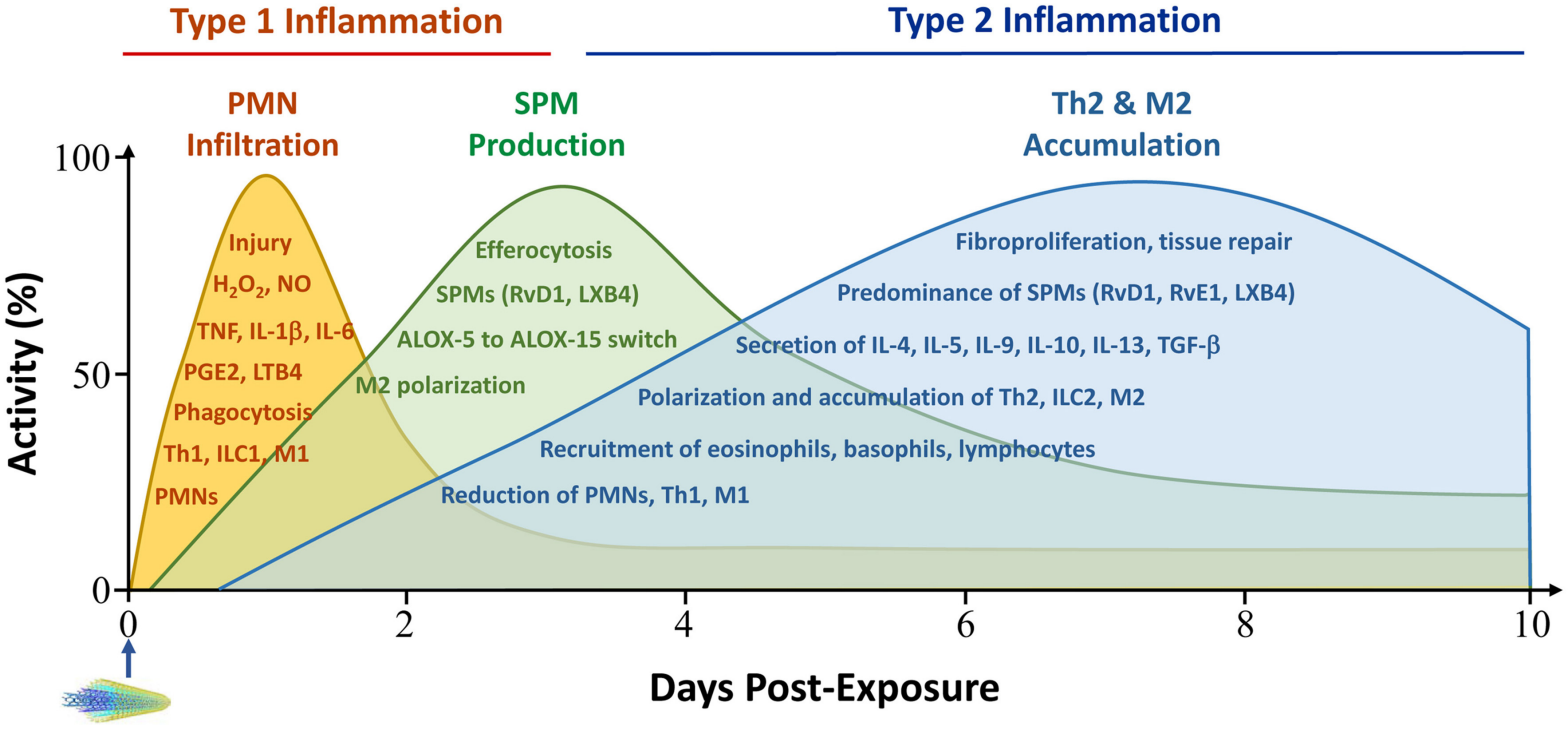
Time-dependent development of type 1 and type 2 inflammation in the acute phase response to particles. Pulmonary exposure to inhaled particulates stimulates the swift recruitment of PMN and other acute inflammatory cells, which is predominated by Th1 and M1-controlled type 1 immune responses. Acute inflammation is reduced in intensity rapidly through active pro-resolution mechanisms, which mainly consist of SPMs, such as lipoxins and resolvins, and type 2 cells and cytokines, such as M2s, IL-10 and TGF-β1. M2s are a major source of SPMs. Accumulation of Th2 and M2 cells begins early, peaks between week 1 and week 2 post-exposure, and extends into the chronic stage of pulmonary lesions. In this context, type 2 inflammation suppresses type 1 inflammation by promoting its resolution and promotes pro-fibrotic and pro-tumorigenic responses by boosting fibroproliferation and the formation of a pro-tumorigenic microenvironment. Major activities during type 1 inflammation, resolution, and type 2 inflammation are listed within corresponding peak areas. PMN infiltration is often measured in the BAL fluid, whereas type 2 cell accumulation manifests in lung tissue. Activity and time scales are for illustration purpose. Multi-walled carbon nanotubes (MWCNTs) are shown to represent particles administered to lungs at time zero. ALOX, arachidonate lipoxygenase; LT, leukotriene; LX, lipoxin; M1, classically activated macrophage; M2, alternatively activated macrophage; NO, nitric oxide; PGE, prostaglandin E; RvD, resolvin D; RvE, resolving E; SPM, specialized pro-inflammatory mediators; Th, T helper; TGF, transforming growth factor; TNF, tumor necrosis factor. Other abbreviations are as described in Figure 1 legend.

Evidence obtained in recent years supports that pulmonary inflammation and fibrosis development induced by inhaled particles is a dynamic and complex process that is dominated by several immune cell-mediated reactions and mechanisms (18, 42, 43). In particular, the recognition of a time-dependent evolvement of type 1 inflammation and its resolution, followed by type 2 inflammation and progression to chronic pathology, has generated new insights into how the pulmonary inflammatory and fibrotic responses to inhaled particles are initiated and propagated in the lung (Figure 2) (18, 42).

### Type 1 Inflammation as the Initial Response to Inhaled Particles

Despite large variations among inhaled particles in their physicochemical properties and pathologic effects, the initial pulmonary response to these particles often follows a common path. This initial response is predominated by acute inflammation that is characterized by rapid recruitment of polymorphonuclear leukocytes (PMNs) and other inflammatory cells, secretion of high levels of proinflammatory cytokines and cytotoxic chemicals, and active phagocytosis of particles by alveolar and airway macrophages. Recent evidence reveals that the acute response to inhaled particles is coordinated by type 1 immune cells, such as M1s, Th1s, ILC1s, and DCs, and type 1 cytokines, such as Th1-derived INF-γ and IL-2, DC-derived IL-12, and M1-derived IL-1β (Table 1) (51–53, 64–68, 78, 79).

**Table 1:** Polarization of immune cells in the immune and inflammatory responses to inhaled particulates.

**Source^**a**^**	**Particle &** **exposure**	**Immune** **response**	**Immune cell polarization, cytokines, & other immune effect**	**Pathologic effect**
Huaux et al. (48) and Barbarin et al. (49)	Silica Mouse, i.t.i.^b^	Type 2	↑ IL-10 in BAL cells and lung tissue. IL-10 deficiency increased acute inflammation, but reduced fibrosis. Transgenic expression of IL-10 boosted silica-induced expression of IL-4 and IL-13, and BAL IgG1 level, and lung fibrotic lesions	Inflammation, fibrosis
Arras et al. (50)	Silica Mouse, i.t.i.	Type 2	↑ Th2-like responses, ↑ IL-4 & IgG1/IgG2a ratio in BAL & ↑ lung fibrosis after 2 and 4 months of exposure. Transgenic expression of IL-9 or injection of IL-9 by i.p. reduces type 2 polarization and fibrosis	Inflammation, fibrosis
Park et al. (51, 52)	MWCNT, SWCNT Mouse, i.t.i.	Type 1, Type 2	MWCNT: ↑ type 1 cytokines IL-1, TNF-α, IFN-γ, and IL-12; ↑ type 2 cytokines IL-4, IL-5, and IL-10; and ↑ IgE, in BAL fluids, time-dependently. SWCNT: ↑ type 1 cytokines IFN-γ and IL-12; and ↑ type 2 cytokines IL-4, IL-5, IL-10, and IL-13, in BAL, time-dependently, which correlated with early fibrosis and subchronic damage	Inflammation, fibrosis
Liu et al. (53)	Silica Mouse, i.t.i.	Th1, Th2, Treg	↑ Th2 & Treg (CD4+ CD25+ Foxp3+) lymphocytes and IL-4, IL-10, and TGF-β levels. Depletion of Treg using anti-CD25 delayed fibrotic progression that correlated with ↑ Th1, but ↓ Th2, responses	Inflammation, fibrosis
Lo Re et al. (54)	Silica Mouse, p.i.	Treg	Silica persistently recruited Treg (CD4+ CD25+ Foxp3+) during lung inflammation and fibrosis. Depletion of Treg ↑ T eff (CD4+ Foxp3-) and production of IL-13, IL-4, IFN-γ, and IL-17A	Inflammation, fibrosis
Wang et al. (55)	MWCNT Mouse, i.t.i.	Type 2	↑ IL-33 (an alarmin in type 2 inflammation), Ccl3 and Ccl11, and Mmp13, which correlate with inflammation, collagen deposition, and granuloma formation	Inflammation, fibrosis
Katwa et al. (56)	MWCNT Mouse, i.t.i.	Type 2	↑ Mast cells, IL-33, and ST2 signaling. Pulmonary and cardiac toxicity of MWCNTs depends on a sufficient population of mast cells and the IL-33/ST2 axis	Pulmonary & cardiovascular effects
Beamer et al. (57)	MWCNT Mouse, i.t.i.	Type 2, ILC2	↑ IL-33 and Th2-dependent cytokines, ↑AHR and eosinophil recruitment, and ↑ ILCs. The effects were dependent on IL-13 signaling and the IL-33/ST2 axis	AHR, inflammation
Ferreira et al. (58)	Silica Mouse, i.n.i.	Type 2	↑ IL-13, IL-13Rα1 and IL-13Rα2, IL-4Rα, granulomatous inflammation and airway hypersensitivity. Exposure of IL-13-PE, an IL-13 based recombinant immunotoxin, reversed the pathologic features of silica	Granuloma, fibrosis, AHR
Freire et al. (59)	Silica Mouse, p.a.	Treg	Induced chronic inflammation, fibrosis, and an immunosuppressive environment, ↑ Treg and expression of TGF-β, FOXP3, and PD1, which increased the incidence and multiplicity of NDMA-induced lung tumor	Tumor promotion
Shvedova et al. (60, 61)	SWCNT Mouse, p.a.	MDSC	↑ recruitment and accumulation of MDSCs in lungs that are associated with increased tumor growth and metastasis in the lung. Depletion of MDSCs or knockout of TGF-β reduces the tumorigenic effect of SWCNT in mouse lung	Cancer growth & metastasis
Ronzani et al. (62)	MWCNT Mouse, i.n.i.	Type 2	↑ Type 2 alarmins TSLP, IL-25, and IL-33, which correlated with elevated responses to HDM, i.e., ↑ total IgG1 and HDM-specific IgG1, influx of macrophages, eosinophils, production of collagen, TGF-β1, mucus, IL-13, eotaxin, and TARC	Allergic-like response to HDM
Rydman et al. (63)	MWCNT Mouse, w.b.i.	Type 2	Rigid rod-like MWCNT stimulated marked eosinophilia, mucus hypersecretion, AHR, and expression of Th2 cytokines in airways, that were in part regulated by master cells as well as alveolar macrophages	Allergic airway response
Meng et al. (64)	MWCNT Macrophage	M1, M2	MWCNTs induced a mixed M1/M2 phenotypes in cultured macrophages.	*In vitro* M1/M2 polarization
Hoppstadter et al. (65)	Silica Macrophage	M1, M2	Comparison of phagocytosis of fluorescent silica and microparticles by M1 or M2-polarized macrophages. M2 polarization is associated with ↑ of particle internalization	*In vitro* phagocytosis
Murthy et al. (66)	Asbestos Human *ex vivo* Mouse, i.t.i.	M1, M2	Alveolar macrophages from patients with asbestosis showed ↑ MARCO, ARG1, and IL-10. Upon exposure to chrysotile, MARCO-/- mice had ↓ fibrosis than wild type; and alveolar macrophages from MARCO-/- mice were M1-like, but those from wild type mice showed M2 phenotypes	Inflammation, fibrosis
Toda and Yoshino (67)	Silica Mouse, i.t.i.	Th1, Th2, Th17	↑ OVA-specific splenocyte proliferation and secretion of Th1, Th2, and Th17 cytokines IFN-γ, IL-2, IL-4, IL-5, and IL-17	OVA-specific response
Labib et al. (68)	MWCNTs Mouse, i.t.i. or p.a.	Type 1, Type 2, Th17	Comparative analyses of 3 microarray data to derive adverse outcome pathway (AOP) for lung fibrosis. Early inflammatory (type 1), Th2 and M2 (type 2), and Th17 responses are implicated in AOP for fibrosis	Inflammation, fibrosis
Dong and Ma (69)	MWCNT Mouse, p.a.	Type 2	↑ Th2 (CD4+ & IL-4+ or IL-13+); ↑ IL-4 and IL-13 mRNA and protein; activation of STAT6 and GATA-3; and ↑ expression of IL-4 target genes on day 7 post-exposure in lungs	Inflammation, fibrosis
Dai et al. (70)	Silica Mouse, i.t.i.	Th1, Th2, Th17, Treg	Knockdown of the Wnt/β-catenin pathway ↓ Treg, ↑ Th17, ↓ Th2, leading to ↑ inflammation and ↓ fibrosis	Inflammation, fibrosis
Huaux et al. (71)	MWCNTs or asbestos Mouse, rat, i.p.	MDSC	Mesotheliomagenic MWCNTs and crocidolite asbestos by i.p. induced accumulation of monocytic-MDSC cells that correlated with the development of peritoneal mesothelioma	Peritoneal mesothelioma
Fatkhutdinova et al. (72)	MWCNTs Human sample	Type 1, type 2	Elevated levels of IL-1, IL-6, TNF-α, TGF-β1, IL-4 in the sputum and serum from workers exposed to MWCNTs	Occupational exposure
Liu et al. (73) and Chen et al. (74)	Silica Mice, i.t.i. Human sample	Breg, Treg, Th2	Silica increased Bregs on days 7, 28, and 56 post-exposure in mice; anti-CD22 attenuated Breg response, which ↑ inflammation, ↓ fibrosis, with ↑ Th1 response, ↓ Treg, Th17, and Th2 responses Patients with silicosis had significantly ↑ serum IL-10, IL-4, IL-5, and IL13; & ↑ Breg (CD19+ CD1d^high^ CD5+ IL-10+), Treg, and Th2 in the blood	Inflammation, fibrosis in mice Silicosis
Duke et al. (75)	MWCNTs Mouse, i.p.	Type 2	Rod-like MWCNT ↑ airway fibrosis, IgE, and TGF-β1, which were exacerbated in STAT1-/- mice	Airway fibrosis
Lebrun et al. (76)	Silica or MWCNTs Mouse, p.a.	MDSC	Both silica and MWCNTs stimulated the acute recruitment of monocytic MDSCs (CD11b+ Ly6C+, CCR2+) into mouse lungs before induced fibrosis. Limiting the MDSCs by using the LysMCreCCR2^loxP/loxP^ mice ↓ TGF-β, Timp1, and collagen in the lung	Inflammation, fibrosis
Maeda et al. (77)	Asbestos Human *ex vivo*	Th17 and Treg	Increased production of IL-17 from CD4+ cells exposed to asbestos ex vivo, indicating altered Th17 and Treg balance associated with immune effects of asbestos in humans	Mesothelioma & asbestosis
Dong and Ma (78)	MWCNT Mouse, p.a.	M1, M2	M1 polarization on day 1 with peak on day 3: ↑ CD86, MHC II, and iNOS; activation of STAT1 & IRF5. M2 polarization on day 3 with peak on day 7: ↑ CD206, CD163, ARG1, Fizz1, and Yam1; activation of STAT6/3 & IRF4	Inflammation, fibrosis
Bao et al. (79)	Silica Rat, i.t.i.	DC, Th1, Th2	↑ DCs slightly during inflammation and significantly during fibrosis. ↑ Th1 and IFN-γ during the inflammatory stage. ↑ Th2 and IL-4 during the fibrotic stage	Inflammation, fibrosis
Rehrauer et al. (80)	Asbestos Mouse, i.p.	M2	↑ Arg expression and Arg+ staining in inflamed mesothelial tissue and mesothelioma, consistent with M2-like tumor-associated macrophage response	Peritoneal mesothelioma
Liu et al. (81)	Silica Rat DC *ex vivo*	DC, Th2	*Ex vivo* exposure of rat DCs to silica induced IL-4 production and Th2 polarization from rat spleen T cells co-cultured with the DCs	*In vitro* Th2 polarization
Benmerzoug et al. (82)	Mouse i.t.i.	Type 2	Silica exacerbated M. tuberculosis infection by enhancing type 2 immunity. ↑ Th2, M2, IL-10, and type 1 IFNs	Tuberculosis

a *References are arranged chronologically. References are combined, if more than one papers from the same authors on the same topic are cited*.

b *AHR, airway hyper-reactivity; AOP, adverse outcome pathway; ARG1, arginase 1; BAL, bronchoalveolar lavage; Breg, regulatory B cell; DC, dendritic cell; FIZZ1, found in inflammatory zone 1; FOXP3, forkhead box P3; GATA-3, GATA-binding protein 3; HDM, house dust mite; IFN, interferon; Ig, immunoglobulin; IL, interleukin; ILC, innate lymphoid cell; iNOS, inducible nitric oxide synthase; i.n.i., intranasal instillation; IRF, interferon regulatory factor; i.t.i., intratracheal instillation; M1, classically activated macrophage; M2, alternatively activated macrophage; MDSC, myeloid-derived suppressor cell; MHC, major histocompatibility complex; MWCNT, multi-walled carbon nanotube; NDMA, N-nitrosodimethylamine; p.a., pharyngeal aspiration; OVA, ovalbumin; PD-1, programed cell death protein 1; p.i., pharyngeal instillation; ST2, IL-1 receptor-like 1; STAT, signal transducer and activator of transcription; SWCNT, single-walled carbon nanotube; TARC, thymus and activation regulated chemokine, CCL17; Th, T helper cell; Treg, regulatory T cell; TSLP, thymic stromal lymphopoietin; w.b.i., whole body inhalation; Yam1, chitinase 3-like 3*.

In type 1 inflammation, polarized Th1, M1, and ILC1 cells and secreted type 1 cytokines coordinate the rapid infiltration of neutrophils and monocytes, and elevated production and secretion of proinflammatory cytokines, such as IL-1β, IL-6, TNF-α, in response to particle exposure. For instance, elevated levels of type 1 cells, type 1 cytokines, and proinflammatory cytokines were detected in the bronchoalveolar lavage (BAL) fluid and lung tissues of rats and mice exposed to silica, asbestos, and CNTs (44–46, 51–53). These acute inflammatory events emerge in hours upon exposure, and peak in 1–3 days, but then decrease rapidly in intensity within a week, which reflects the self-limiting nature of acute type 1 inflammation (44–46). Elevated levels of IL-1β, IL-6, and TNF-α were also detected in the sputum and serum collected from workers exposed to MWCNTs (72). These findings support that the early acute inflammatory response to inhaled particles in the lung is predominated by type 1 inflammation, which is governed primarily via the polarization of type 1 immune cells.

### Polarization of Type 1 Immune Cells

#### DCs, ILC1s, and Th1s

The initiation of type 1 inflammation involves the activation of DCs and ILC1s. Activated DCs and ILC1s produce IL-12 and IL-18 that induce the polarization of Th1 (CD4+ IFN-γ+) lymphocytes from naïve CD4+ Th0 cells. Th1 and ILC1 cells secrete IFN-γ and TNF-α that stimulate the polarization of macrophages toward an M1 phenotype, a major cellular event in the initiation and amplification of type 1 inflammation. A recent study reveals that exposure of rats to silica by intra-tracheal instillation increased the number of Th1 cells and the levels of IFN-γ and IL-12 in the lung, which were likely to be stimulated by increased recruitment of DCs in the early inflammatory phase of the pulmonary response (79). In a separate study, exposure to amorphous silica nanoparticles enhanced the polarization of Th1 lymphocytes and the production of IFN-γ in ovalbumin (OVA)-sensitized mice (67). Exposure of mice to MWCNTs or single-walled CNTs (SWCNTs) elevated the levels of IL-12 and IFN-γ, indicating the activation of DCs, ILC1s, and Th1 lymphocytes by inflammatogenic CNTs (51, 52).

#### M1 Macrophages

M1 macrophages are primary effector cells in type 1 inflammation. M1s are characterized by their high capacity of phagocytosis of microbes and particles and their production of large quantities of type 1 inflammatory mediators. Type 1 mediators include proinflammatory cytokines, such as IFN-γ, IL-1, IL-6, IL-12, IL-23, and TNF-α, bactericidal agents, such as NO, and matrix-remodeling enzymes, such as matrix metallopeptidases (MMPs). Together, these cellular and molecular events enable the enhanced engulfment of particles and rapid evolvement of type 1 inflammation. In an animal study, pulmonary exposure to MWCNTs increased the number of M1 macrophages in mouse lungs by day 1 that reached a peak level on day 3 post-exposure (78). These M1 macrophages had elevated levels of CD86, MHC II, and inducible nitric oxide synthase (iNOS). Expression of these M1 markers involves the activation of two transcription factors, i.e., the signal transducer and activator of transcription (STAT) 1 and the interferon regulatory factor (IRF) 5 (78). Chrysotile asbestos exposed to mouse lungs induced the polarization of AMs toward an M1 phenotype in the absence of the macrophage receptor with collagenous structure (MARCO) (66). *In vitro* exposure of cultured macrophages to MWCNTs stimulated the differentiation of the cells to a mixed population of M1 and M2 cells (64).

### Particle Sensing and Signaling Through PRRs in Type 1 Inflammation

The mechanism by which immune cells sense particulates and how these signals are transduced to initiate and amplify type 1 inflammation involve complex cellular and molecular mechanisms that are not well understood at the present time. It is believed that PRRs of immune cells—in particular, AMs, DCs, and epithelial cells—play a major role in the recognition of particles and their associated signals. In this regard, MARCO at the surface of AMs appears to mediate the binding and uptake of crystalline silica particles and perhaps asbestos fibers as well by macrophages (66, 83). The NOD-like receptor family, pyrin domain containing 3 (NLRP3) inflammasome is a cytoplasmic multi-protein oligomer complex that regulates acute inflammation by controlling the proteolytic cleavage, maturation and secretion of IL-1β and IL-18 (84). NLRP3 senses and integrates a wide range of exogenous and endogenous signals, such as various DAMPs released from damaged cells. Inorganic particulates, including silica, asbestos, monosodium urate (MSU) crystals, cholesterol crystals, and nanoparticles, have been shown to activate the NLRP3 inflammasome in cultured macrophages *in vitro* and in lung tissues *in vivo* (85–87). The mechanism by which these particulates activate NLRP3 inflammasome remains unclear but may involve the production of ROS induced by exposure to particles. This NLRP3/IL-1β pathway is clearly required for the acute inflammatory response to particles in the lung, as suppression of the pathway by knocking out the gene encoding IL-1 receptor (IL-1R1) or inhibiting IL-1R1 signaling using IL-1R1 antagonist anakinra attenuated neutrophilia in the lung (85, 86). At the level of gene transcription, induction of many type 1 cytokines and growth factors, such as TNF-α, IL-1β, and IFN-γ, involves the transcription factor nuclear factor kappa-light-chain-enhancer of activated B cells (NF-κB). A recent study has delineated the signaling pathway of NF-κB activation in mouse lungs after exposure to MWCNTs, which provides a mechanistic framework for *in vivo* analysis of gene regulation in pulmonary type 1 inflammation induced by inhaled particulates (88).

### Type 1 Inflammation and Particle Clearance

A primary purpose of the type 1 inflammatory response to inhaled particles is to clear the particles from the lung. Unlike microbial clearance where both neutrophils and macrophages engulf and kill invading pathogens, the pulmonary clearance of inorganic particles is mediated mainly through phagocytosis by AMs and airway and interstitial macrophages. These macrophages exhibit M1 phenotypes and have a high efficiency and plasticity with respect to the nature and dimensions of particles and fibers that are engulfed. In contrast, neutrophils appear to be incapable of, or very inefficient in, phagocytosing inorganic particles, even though they are recruited to the affected lung tissue site rapidly and in large numbers. Because inorganic particles cannot be digested and degraded by phagocytes quickly, engulfed particles are transported to local lymph nodes and drained into the blood circulation via lymphatic vessels. Particles deposited in the airways are also discharged through the muco-ciliary escalator of the airway epithelium, which is facilitated by type 1 macrophages and inflammatory secretions in the airways.

Despite these elaborate clearance mechanisms in the lung, a major portion of inhaled particles retain in the interstitial space. Because engulfed particles cannot be digested by macrophages, phagocytosis of particles results in the apoptosis or other forms of cell death of M1 macrophages. It is believed that a sustained cycle between phagocytosis of particles and programmed death of macrophages in lung tissue leads to persistent inflammatory events and cell death at the site of particle deposition, which in turn stimulates interstitial fibrosis and formation of granulomas to localize and enclose deposited particles and fibers.

Some inhaled fibers like asbestos and certain carbon nanotubes are capable of translocating from the lung to the pleural cavity to cause pleural inflammation, plaques, and mesothelioma. Fibers and nanotubes that enter the pleural cavity are cleared from the pleural space through phagocytosis by M1 macrophages or via drainage through parietal pleural stomata openings to local lymph nodes. These clearance mechanisms have considerable limitations on the size of the fibers that can be cleared from the pleural cavity, which provides a mechanistic explanation for the fiber-length relationship for induction of mesothelioma by asbestos and nanotube fibers (11, 89, 90).

### Resolution of Type 1 Inflammation

During type 1 inflammation, large amounts of cytotoxic agents, including ROS and RNS, are produced and released from macrophages due to frustrated phagocytosis, or from apoptotic neutrophils and damaged tissue, which can lead to substantial damage to host cells and tissue structure (91). Therefore, type 1 inflammation is generally contained in space and time by means of active resolution following the removal or reduction of pathogens and other instigators. Resolution of inflammation avoids excessive tissue damage from type 1 inflammatory mediators and bactericidal agents (92). During resolution, infiltration of inflammatory cells is reduced, neutrophils undergo apoptosis, and macrophages engulf and degrade apoptotic neutrophils and cell debris through a process called efferocytosis. Together, these resolution events clear the inflammatory site and promote the repair of damaged tissue and return of homeostasis. Resolution is mediated through an array of chemical, protein, and cell signals, in particular, SPMs (Figure 2) (42, 47, 93). Because most particles and fibers cannot be digested by phagocytes, resolution of inflammation induced by inhaled particles is often incomplete, which stimulates the chronic progression of inflammation, interstitial fibrosis, granuloma formation, and tumorigenesis at the site of particle deposition. A recent study showed that exposure to fibrogenic MWCNTs at a low dose or fullerene C60 (C60F, a PSLT type of nanoparticles) at a high dose stimulates the production of SPMs in the BAL fluid, which correlated with the reduction of neutrophil numbers in BAL fluids during resolution (94).

When exposure to particles persists and overwhelms the clearance capacity, lung inflammation cannot be resolved completely. Unresolved type 1 inflammation propagates and causes further damage to lung tissue through cytotoxic agents, such as NO, and matrix-degrading enzymes, such as MMPs, resulting in the chronic progression of inflammation. Chronic inflammation is marked by a progressive shift in the type of cells present at the site of inflammation, often shown as increased mononuclear cells. In both human and rodent lungs exposed to particles like silica and fibrogenic CNTs, chronic inflammation is manifested as granulomatous inflammation. It is increasingly recognized that a failed or incomplete resolution of type 1 inflammation in the lung is a critical early step in the pathogenesis of chronic disease conditions induced by inhaled particles. Nonetheless, the research on the resolution of inflammation induced by inhaled particles is at its early stage and would await further detailed investigation before its role in particle pathogenesis can be fully appreciated.

## Type 2 Inflammation in the Pulmonary Response to Particles

Type 2 inflammation is characterized by the polarization of Th2 cells, ILC2s, and M2 macrophages, and the secretion of type 2 cytokines IL-4, IL-5, IL-9, IL-10, and IL-13 (Figure 1). The traditional view of type 2 immune reactions is such that these distinct repertoire of immune cells and cytokines are optimized for host resistance against helminth infection. Dysregulated or heightened type 2 inflammation may cause allergic reactions and conditions, such as asthma and anaphylaxis (30). Besides these well-established functions, recent studies reveal that type 2 cells and mediators are involved in diverse biological processes, including tissue repair, fibrosis, metabolic control, and tumorigenesis and metastasis (93). Moreover, ILC2s were shown to secrete large amounts of type 2 cytokines, which provides a mechanistic explanation to the activation of type 2 inflammation in the absence of apparent antigenic stimulation and antigen-specific immunity (34).

Upon exposure to inhaled particulates and immediately following the onset of type 1 inflammation, there is increasing and persistent accumulation of macrophages in lung tissue (Figure 2). Morphologically, the particle-induced lesions are characterized by the formation of fibroproliferative and inflammatory foci in the parenchyma that are composed of particle-laden macrophages, infiltrated neutrophils, proliferating fibroblasts and myofibroblasts, and clusters of particles and fibers, all of which are intermingled with bundles of collagen fibers. The fibrotic matrix remodeling and formation of fibroproliferative and inflammatory foci likely represent the early stage fibrosis and granuloma formation (45, 46, 95). These tissue changes occur during the 1st week of exposure and peak between the 1st and 2nd week (69, 96, 97). The pathological progression following type 1 inflammation is accompanied by elevated expression and secretion of type 2 cytokines IL-4 and IL-13, and the polarization of Th2 lymphocytes and M2 macrophages, which signify the development of type 2 inflammation (69, 78). These findings reveal that type 2 inflammation evolves during the resolution phase of type 1 inflammation, prior to the chronic progression to fibrosis and tumor development (42).

### Type 2 Mediators

#### Type 2 Cytokines

A large body of literature documented the induced expression of type 2 cytokines and mediators at protein and mRNA levels in BAL fluids and lung tissue of different species by inhaled particles, fibers, and nanoparticles (Table 1). As an example, silica induced type 2 cytokines, such as IL-10, IL-4, IL-13, or IL-9, in the BAL fluid and lung tissues of mice, and increased the level of IgG1 in the BAL fluid (48–50, 53, 58). MWCNTs and SWCNTs each stimulated the production of type 2 cytokines IL-4, IL-5, IL-10, or IL-13, and elevated the level of IgE in the BAL fluid in mice (51, 52). A genome-wide microarray analysis identified elevated expression of type 2 cytokines IL-4 and IL-13, along with a panel of their downstream target genes, i.e., Il4i1, Chia, and Ccl11/Eotaxin, in mouse lungs exposed to fibrogenic MWCNTs. Induction of the genes and proteins occurred between the 1st and the 2nd week with a peak at day 7 upon a single exposure (69). Exposure of rats to silica induced the expression of IL-4 during the fibrotic stage of the pulmonary response (79). Patients with asbestosis have elevated expression of IL-10 in their AMs (66), whereas patients with silicosis showed increased levels of IL-10, IL-4, IL-5, and IL13 in their serum (74). Elevated levels of IL-4 and IL-5 were detected in the sputum and/or the serum of workers exposed to MWCNTs (72).

#### Alarmins

Alarmins are groups of immune mediators that are released from damaged or diseased cells and serve as DAMPs to stimulate immune reactions in response to distinct pathogens and sterile threats. Pulmonary exposure to instigators of type 2 immunity and inflammation, such as helminth infection and allergen exposure, induce the production of IL-33, IL-25, and thymic stromal lymphopoietin (TSLP) from lung epithelial and endothelial cells. These alarmins then recruit and activate a set of innate immune cells, such as eosinophils, mast cells, basophils, ILC2s, DCs, and M2s, which initiate type 2 responses by providing the initial production of IL-4 and IL-13 to induce Th2 polarization. Recent evidence showed that exposure to MWCNTs in mice induced the expression of IL-33 in the BAL fluid, the lung tissue, AMs, and type II alveolar epithelial cells (55–57, 62). Blocking IL-33 signaling with antibodies against IL-33 receptor, i.e., IL-1 receptor like 1 (ST2), or using mice with mast cells lacking ST2, significantly reduced type 2 immune responses, demonstrating a critical role of the IL-33/ST2 axis in the initiation of type 2 inflammation upon particle exposure (56, 57). An unbiased genome-wide array analysis identified TSLP as an inducible gene by MWCNTs in mouse lungs that corresponded to the occurrence of type 2 inflammation (69). MWCNTs increased the levels of TSLP, IL-25, and IL-33 in mouse lungs, which correlated with an elevated allergic response to house dust mite (HDM), a commonly used inducer of asthmatic-like inflammation in the airway (62).

#### TGF-β1

TGF-β1 is a key mediator of type 2 responses and downstream outcomes (93). TGF-β1 is produced by type 2 cells, mainly, M2 macrophages, during type 2 inflammation. TGF-β1 promotes the transformation of fibroblasts to myofibroblasts, a key cellular event in fibrosis development, by increasing the transcription of α-smooth muscle actin (α-SMA) via the Sma and Mad related family (Smad)-dependent gene transcription (95, 97). TGF-β1 is also a potent negative regulator of Th1-mediated type 1 inflammation (88). In this connection, TGF-β1 promotes the resolution of acute inflammation but stimulates the progression to chronic inflammation and fibrosis (42, 98). Elevated expression of TGF-β1 was detected in mice exposed to silica (53, 59). Mice exposed to SWCNTs or MWCNTs had elevated levels of TGF-β1 (61, 62). Fibrogenic MWCNTs induced the expression of TGF-β1 protein in mouse lungs during both the acute and chronic phases of the inflammatory and fibrotic responses (97). Recent evidence from a human study revealed significantly elevated level of TGF-β1 in the serum of workers exposed to MWCNTs (72).

### Polarization of Type 2 Immune Cells

#### Th2s

Th2s are CD4+ T lymphocytes that control the initiation and amplification of type 2 immune responses by secreting type 2 cytokines IL-4, IL-5, IL-9, IL-10, IL-13, and IL-25 (Figure 1). Downstream effector cells of Th2s include eosinophils, basophils, mast cells, B cells, macrophages, and fibroblasts. Polarization of Th2s from naïve CD4+ Th0 lymphocytes is initiated by IL-4 and IL-2 and is amplified by IL-4 and IL-13. As discussed above, alarmins released from injured cells stimulate the initial production of IL-4 and IL-13 from ILC2s and other cells, which, in turn, triggers the polarization of Th2s. IL-4 and IL-13 bind to their receptors on the cell surface to activate Th2 polarization and the production of type 2 cytokines and their target genes, which are mediated through the activation of STAT6 and GATA-binding protein 3 (GATA-3) (42, 69).

Induction of Th2 polarization was observed in mouse lungs exposed to fibrogenic MWCNTs at days 1, 3, 7, and 14 post-exposure, with increased levels of CD4+ IL4+ or CD4+ IL13+ lymphocytes (69). Polarization of the Th2 cells was accompanied by increased secretion of IL-4 and IL-13, activation of STAT6 and GATA-3, and elevated levels of IL-4 target genes, such as Il4il, Chia, and Ccl11. Exposure of rats to silica increased the number of Th2 cells alongside elevated expression of IL-4 and IL-10, which was boosted by depletion of Treg lymphocytes (53). Knockout of STAT6 in mice appeared to block Th2 polarization induced by MWCNTs as shown by loss of induction of IL-5 in STAT6 knockout mice compared to wild type (86). Silica also increased the number of Th2 cells in fibrotic lung tissues of rats (79). Patients with silicosis had elevated level of Th2 cells (74). *Ex vivo* exposure of rat DCs to silica induced IL-4 production and Th2 polarization from rat spleen T cells co-cultured with the DCs (81).

#### M2 Macrophages

M2 macrophages are key effector and regulator cells in type 2 inflammation. Among their many functions, M2s suppress Th-1 mediated type 1 inflammation and promote tissue repair, fibrosis, and tumorigenesis (98). M2s can be separated into several subgroups phenotypically, including M2a, M2b, M2c, and M2d. Among these subtypes, M2a macrophages are activated by IL-4 and IL-13 and regulate tissue repair and suppression of type 1 inflammation through IL-10 and TGF-β. Therefore, M2a macrophages are likely to be involved in the pulmonary response to inhaled particles. Notably, polarization of M2s is highly plastic with respect to their phenotypes and functions and can be induced by a range of stimuli and microenvironmental cues. Polarization of M2s is mediated through STAT6- and IRF4-mediated gene transcription.

Direct evidence of M2 polarization by inhaled particles was provided in a recent study on mice exposed to fibrogenic MWCNTs. MWCNTs induced time-dependent expression of M2 surface markers MRC1 (CD206) and hemoglobin scavenger receptor (CD163), and functional M2 markers ARG1, found in inflammatory zone 1 (FIZZ1), resistin-like molecule α (RELMα), and chitinase 3-like 3 (YM1), in the lung and lung F4/80+ macrophages *in vivo* (78). Polarization of M2s involved the phosphorylation of STAT6 and induction of IRF4 in lung macrophages. A separate study showed that exposure of mice to asbestos fibers elevated the expression of ARG1 in inflamed mesothelial tissue and mesothelioma, indicating activation of tumor-associated M2 macrophages (80). Direct exposure of macrophages to MWCNTs *in vitro* induced a mixed phenotype of M1 and M2 cells. This result suggests that that CNTs can stimulate M2 polarization from naïve macrophages directly, but induction of M2 requires additional signals for it to become a predominant phenotype, which was absent in the *in vitro* system, but is provided in the lung by type 2 immune cells and injured epithelial cells (42, 64).

#### ILC2s

ILCs are a group of innate helper lymphocytes that derive from the common lymphoid progenitors, but lack antigen-specific B or T cell receptors. Polarization of ILC2s is stimulated by type 2 alarmins. Activated ILC2s secrete type 2 cytokines, including IL-4, IL-5, IL-9, and IL-13, to boost type 2 inflammation. Notably, ILC2s are now recognized as a type of highly responsive, early effectors in type 2 inflammation. Activated ILC2s serve as an important source for the early production of IL-4 that stimulates the initiation of Th2 polarization. Moreover, ILC2s appear to have the capacity to secret large amounts of type 2 cytokines even without the presence of adaptive immunity, which can in part account for the activation of type 2 inflammation by a broad range of stimuli (34).

MWCNTs induced airway hyperreactivity (AHR) in mice, which was IL-33-dependent and was accompanied by elevated expression of IL-5 and CCL11 and recruitment of eosinophils (57). These allergic-like responses to MWCNTs appeared to be independent of T and B cells; but were correlated with the recruitment of ILCs that are lineage negative (F4-80, CD3, CD4, CD8, CD11b, CD11c, CD19, Gr-1, TER119, FcεR1), and are CD45+ Sca-1+ IL-7Rα+ and ICOS+. These findings suggest that MWCNTs recruit ILCs to mediate the recruitment of eosinophils and the secretion of certain type 2 cytokines during the development of airway AHR and allergic inflammation.

### Type 2 Immune Mechanisms in the Resolution of Acute Inflammation and Progression of Chronic Pathology

The accumulation of type 2 cells and cytokines begins during the resolution phase of type 1 inflammation. Type 2 inflammation reaches a peak at the junction of the acute-to-chronic transition and extends into the chronic phase of lung pathology (Figure 2). This time-dependent evolvement from type 1 to type 2 inflammation and then to chronic pathology likely reflects the nature and consequence of the interaction between inhaled particles and the pulmonary immune system. At the fundamental level, this interaction is set to clear particles from the lung through type 1 inflammation, but often fails to do so, because inorganic particles generally cannot be digested by M1 macrophages. As a result, a major portion of inhaled particles retain in the lung parenchyma, resulting in persistent tissue injury, a pathologic condition that is well suited for type 2 inflammation to occur and propagate as a host response to persistent injury. In this context, type 2 inflammation suppresses type 1 inflammation to mitigate tissue damage through the polarization of M2s that phagocytose dead neutrophils and particles, and the secretion of IL-10 and TGF-β1 that inhibits the continued polarization of Th1 and M1. Type 2 inflammation also promotes the active resolution of type 1 inflammation. M2 macrophages have been identified as a major source of SPMs that mediate the active resolution of acute inflammation (94, 99). Mechanistically, polarization of M2s induces the expression of 15-lipoxigenase (ALOX15), a key enzyme in the synthesis of major SPMS, i.e., resolvin Ds (RvDs) and lipoxins; but suppresses the expression of arachidonate 5-lipoxigenase-activating protein (ALOX5AP), a major regulator protein for the synthesis of proinflammatory lipid mediators, such as leukotriene B4 (LTB4) and prostaglandin E2 (PGE2) (94). This metabolic switch in the synthesis of proinflammatory to pro-resolving lipid mediators is key to active resolution of inflammation mediated through the reduction of inflammatory infiltration and increase of efferocytosis of dead cells in particle-exposed lungs.

As type 1 inflammation is resolved, the pulmonary response converts to a fibroproliferative and inflammatory response characterized by the formation of fibrotic foci, which are composed of macrophages with engulfed particles and fibers, PMNs, some other inflammatory cells, and fibroblasts and myofibroblasts, intermingled with bundles of collagen fibers (Figure 2). Among type 2 mediators, TGF-β1 released from M2s plays a major role in the fibroproliferative response by promoting the polarization of fibroblasts into myofibroblasts, which secrets copious amounts of collagen proteins and mediate the contraction of fibrotic tissues. Morphologically, fibroproliferative foci may represent an early stage granulomatous lesion that later develops into granulomas. Increasing evidence reveals that type 2 inflammatory lesions stimulate the development of fibrosis, granulomatous inflammation, airway hyperreactivity and fibrotic remodeling, and malignancy (18, 42). It is known that STAT1 mediates M1 polarization, whereas STAT6 is required for the polarization and activation of Th2s and M2s. Mouse studies showed that loss of STAT1 reduced type 1 inflammation but enhanced type 2 inflammation and fibrosis induced by fibrogenic MWCNTs (75). On the other hand, knockout of STAT6 in mice significantly reduced pulmonary fibrosis, which correlated with reduced type 2 cytokine IL-5, at day 28 post-exposure to MWCNTs (86). In studies on type 2 alarmins, mast cells and the IL-33/ST2 signaling axis have been shown to be required for CNT-induced AHR, lung fibrosis, granuloma formation, and reduction of lung functions (56, 57). Exposure of mice with fiber-like MWCNTs by inhalation induced allergic airway inflammation in healthy mice, characterized by eosinophilia, mucus hypersecretion, AHR, and the expression of Th2-type cytokines. In part, these responses were mediated through airway mast cells (63). Exposure of mice to silica exacerbated Mycobacterium tuberculosis infection by upregulating type 2 immunity with elevated levels of Th2s, M2s, and IL-10 (82).

## Other Immune Cell-Mediated Responses to Inhaled Particles

In addition to Th1-associated type 1 inflammation and Th2-associated type 2 inflammation, several other immune cell-mediated responses have been observed in the lung exposed to particles, fibers, or ENMs (Figure 1). Recent evidence supports that these responses play important roles in disease development induced by inhaled particles. On the other hand, these responses overlap with the dichotomous type 1 and type 2 inflammation in time and space. Moreover, there has not been sufficient evidence to support that these responses govern a specific immune outcome in particle-exposed lungs. Therefore, these responses are not considered as unique types of immunity or inflammation, but as immune cell-mediated reactions that regulate and modulate the pathologic development of chronic outcomes induced by particles in the current review.

### Th17-Mediated Response

Th17 lymphocytes are a new subset of pro-inflammatory, helper CD4+ T cells defined by their production of IL-17, also known as IL-17A (Figure 1) (100). Cytokines secreted by Th17s include IL-17A, IL-17F, IL-21, and IL-22. The polarization of naïve T cells to Th17s can be triggered by TGF-β, IL-6, IL-21, and IL-23, and requires the activation of transcription factors STAT3, the retinoic acid receptor-related orphan receptors γ (RORγ) and α (RORα). Therefore, Th17s are developmentally distinct from Th1 and Th2 cells. Th17s play important roles in maintaining mucosal barriers and in pathogen clearance at mucosal surfaces against bacterial and fungal infections. As a potent proinflammatory cytokine, IL-17 amplifies ongoing inflammation by inducing the expression of TNF-α, IL-1β, and IL-6 in epithelial and endothelial cells, as well as keratinocytes, synoviocytes, fibroblasts, and macrophages. Th17s have been implicated in autoimmune and inflammatory disorders. Because of their importance in mucosal barrier immunity against pathogens, Th17-mediated immune reactions are sometimes called type 3 immunity (30).

Several studies demonstrated the induction of Th17 cytokines and Th17 polarization by particulates in the lung. Elevated expression of IL-17 was observed in mouse lungs exposed to silica and the induction was suppressed by Tregs (54). In this model, IL-17 contributed to an increased fibrotic response. Exposure of amorphous silica nanoparticles in mice challenged with OVA showed enhanced OVA-specific immune responses, including an elevated Th17-mediated response and increased production of IL-17 (67). In a separate study, silica was shown to elevate Th17s in mouse lungs, which was enhanced by knockdown of the Wnt/β-catenin pathway. These changes correlated with increased inflammation in the early stage, but decreased fibrosis in the later stage (70). Comparative analyses of three separate sets of microarray data obtained from mice exposed to MWCNTs implicated the Th17 response in the adverse outcome pathway (AOP) for lung fibrosis development (68). In addition, CD4+ T cells from patients exposed to asbestos showed an altered balance between Th17 and Treg responses with elevated Th17 reactions upon *ex vivo* exposure of the cells to chrysotile fibers (77). Therefore, exposure to inhaled particulates increases Th17 responses that are associated with altered inflammation and/or fibrosis in the lung exposed to particles. In these examples, increased Th17 levels and functions are often associated decreased Treg responses, consistent with a mutual inhibitory nature of the interactions between Th17s and Tregs.

### Treg-Mediated Immunosuppressive Response

Tregs are a group of T cells critical for the maintenance of immune tolerance (101). As immunosuppressive cells, Tregs suppress or downregulate the induction and proliferation of effector T cells. Tregs consist of several forms, among which the most well-understood are those that are CD4+ CD25+ and FoxP3+. Vitamin A and TGF-β promote T cell polarization toward Tregs as opposed to Th17 cells. Tregs suppress auto-reactive T cells that have escaped the process of negative selection in the thymus. This immunosuppressive function of Tregs is key to the prevention of autoimmunity (102). A genetic deficiency in Tregs causes the development of a severe autoimmune syndrome termed immune dysregulation, polyendocrinopathy, enteropathy X-linked (IPEX) syndrome in humans. Pathogens may utilize Tregs to suppress host immune functions. On the other hand, Tregs are involved in stopping immune responses after invading pathogens have been eliminated. In addition to these immunosuppressive activities, Tregs suppress tumor immunity and high numbers of Tregs in the tumor microenvironment is indicative of a poor prognosis. Additionally, Tregs may promote tissue repair and regeneration (103). The mechanism by which Tregs regulate other immune cells is not completely understood, but would include the production of immune suppressive cytokines, such as IL-10, IL-35, and TGF-β1. Tregs can also induce other types of cells to produce IL-10.

Elevated Treg-mediated immunoregulatory responses were observed in the pulmonary response to inhaled particulates. Pulmonary exposure of mice to silica elevated the level of Treg lymphocytes (CD4+ CD25+ FoxP3+), along with increased levels of Th2 cells and type 2 cytokines, in hilar lymph nodes (HLNs), the spleen, and BAL fluids (53). Moreover, depletion of Tregs by using anti-CD25 antibodies increased the intensity of inflammation in the early stage of the pulmonary response, evidenced by enhanced infiltration of inflammatory cells. On the other hand, the progression toward fibrosis in the late stage was delayed. These phenotypes correlated with increased expression of cytotoxic T lymphocyte-associated protein 4 (CTLA-4, CD152) in the inflammatory stage and elevated secretion of IL-10 and TGF-β in the fibrotic stage. In this scenario, depletion of Tregs altered the balance between Th1 and Th2 responses, enhancing the Th1 response in the early stage and delaying the polarization of Th2 phenotype for fibrosis in the late stage.

Pharyngeal instillation of silica persistently recruited Tregs (CD4+ CD25+ FoxP3+) to mouse lungs during lung inflammation and fibrosis (54). Selective depletion of Tregs by using the DEREG mice, which express the diphtheria toxin receptor under the control of the foxp3 gene, resulted in the enhanced accumulation of CD4+ T eff cells and IL-4-driven pulmonary fibrogenesis, demonstrating that Tregs control T eff cell functions during inflammatory fibrosis induced by silica. In a mouse model of lung carcinogenesis by N-nitrosodimethylamine (NDMA), silica exposure by pharyngeal aspiration induced chronic inflammation, fibrosis, and an immunosuppressive microenvironment in the lung, marked by increased Tregs and elevated expression of TGF-β, FoxP3, and programed cell death protein 1 (PD1), which resulted in an increased incidence and multiplicity of lung tumors in the presence of NDMA (59). The Wnt/β-catenin pathway may play an important role in the regulation of T eff cells through Tregs, because knockdown of Tregs by using β-catenin shRNA in mice significantly aggravated silica-induced lung inflammation at the early stage but attenuated the fibrosis at the late stage (70). Blockade of the Wnt/β-catenin pathway suppressed Treg responses, while elevating T17 responses, which increased inflammation but decreased Th2 response, leading to attenuated fibrosis. In a separate study, CD4+ lymphocytes from patients exposed to asbestos fibers exhibited altered functions of Tregs. Exposure of the CD4+ cells *ex vivo* showed an altered balance between Treg and Th17, with a decreased Treg response but elevated T17 response (77).

### Breg-Mediated Immunosuppressive Response

Regulatory B cells (Bregs) are a small group of B cells that participate in the immunomodulation and suppression of immune reactions (104). A major mechanism of immune suppression by Bregs is the production of anti-inflammatory cytokine IL-10. IL-10 strongly inhibits or suppresses inflammatory reactions mediated by T cells, especially the Th1-mediated reactions. Bregs also produce TGF-β, which also suppresses inflammation. Bregs have been implicated in disease models of inflammation, autoimmune dysfunction, transplantation reactions, and anti-tumor immunity.

Recent evidence supports a role of Bregs in the regulation of the inflammatory and fibrotic responses to silica in animals and in patients with silicosis. Exposure of mice to silica increased the level of Bregs (CD19+ IL10+) on days 7, 28, and 56 post-exposure in HLN and the spleen in mice, which correlated with induced inflammation in the early stage and fibrosis in the late stage (73). Intraperitoneal injection of anti-CD22 antibodies attenuated the silica-induced Breg response, resulting in elevated inflammation but reduced fibrosis. These pathological alterations correlated with an increased Th1 response, but decreased Treg-, Th17-, and Th2-mediated immune responses. In a different study, patients with silicosis were found to have elevated level of plasma IL-10 through a protein array screening of plasma cytokines. The patients also showed increased levels of IL-10-producing Bregs (CD19+, CD1d^high^, CD5+, IL-10+) in their peripheral blood, in comparison with subjects under surveillance and healthy workers (74). Treg cytokines (TGF-β and IL-10) and Th2 cytokines (IL-4, IL-5, and IL-13), but not Th1 cytokines (IFN-γ, IL2, and IL-12) and pro-inflammatory cytokines (IL-1β, IL-6, and TNF-α), were found to be increased in the sera of patients with silicosis. Together, these findings support the notion that Bregs are induced by silica to maintain Tregs and to regulate the balance among Th1, Th2, and Th17 toward type 2 inflammation, which boosts fibrosis in the lung.

### MDSC-Mediated Immunosuppression in Particle-Induced Pathologic Conditions

MDSCs are a heterogeneous group of myeloid cells that are discriminated from other myeloid cells by their strong immunosuppressive, rather than immunostimulatory, activities (105). The suppressor function of MDSCs lies in their ability to inhibit the proliferation and function of T cells, natural killer cells (NKs), DCs, and macrophages. Mouse MDSCs express either high levels of CD11b and Ly6C—i.e., monocytic-MDSC (M-MDSC); or CD11b, LY6C and high levels of LY6G—i.e., granulocytic-MDSC (G-MDSC). MDSCs are polarized as a result of perturbed myelopoiesis caused by various pathological conditions. For example, in chronic inflammation or cancer, myeloid differentiation is skewed toward the expansion of MDSCs (106). When MDSCs infiltrate inflammatory tissue and tumors, they inhibit immune responses by suppressing T cells, NK cells, and macrophages. MDSCs were implicated in immune regulation and disorders, such as cancer, chronic inflammation, infection, autoimmune diseases, trauma, and graft vs. host reactions.

Pulmonary exposure to SWCNTs in mice accelerated the metastatic establishment and growth of Lewis lung carcinoma (LLC) in the lung, which correlated with the increased local and systemic accumulation of MDSCs (60). Depletion of the MDSCs by using anti-Gr-1 antibodies prevented the SWCNT-induced up-regulation of MDSCs in the lung and the spleen and blocked the SWCNT-mediated acceleration of the formation and growth of lung metastases of LLC. In a separate study, SWCNTs were shown to upregulate TGF-β1 production by tumor-activated MDSCs, which promoted an immunosuppressive, pro-tumorigenic microenvironment in mouse lung for the growth and metastasis of LLC (61). Accordingly, TGF-β1 deficiency completely abrogated the CNT-induced acceleration of cancer growth in the lung. Therefore, TGF-β1 produced by activated MDSCs was likely to be responsible for the suppression of T cell activation and proliferation in this LLC model of tumor growth and metastasis promoted by SWCNTs.

Administration of mesotheliomagenic MWCNTs or crocidolite asbestos to rats by intraperitoneal injection induced an early and selective accumulation of M-MDSCs in the peritoneal cavity (71). Peritoneal M-MDSCs persisted during the development of peritoneal mesothelioma in mesotheliomagenic MWCNT-treated rats. In contrast, M-MDSCs were only transiently recruited upon non-carcinogenic CNT injection. These findings support an important role of M-MDSC induction in the development of mesotheliomas induced by MWCNTs and asbestos. The role of M-MDSCs in particle-induced lung inflammation and fibrosis was investigated in mice exposed to fibrogenic MWCNTs or silica (76). Both silica and MWCNTs stimulated the acute recruitment of M-MDSCs (CD11b+ Ly6C+ CCR2+) into mouse lungs before the development of fibrosis. Limiting the MDSCs by using the LysMCreCCR2^loxP/loxP^ mice decreased the levels of TGF-β, Timp1, and collagen in the lung. These findings suggest that M-MDSCs contribute to lung fibrosis induced by fibrogenic particulates by fostering a non-degrading collagen microenvironment.

## Integration of Immune Mechanisms in Particle Pathogenesis

Pulmonary exposure to inhaled particulates potentially causes a range of diseases that are frequently chronic and progressive, leading to severe outcomes. Evidence obtained in recent years supports that the progression of the pulmonary response to particulates toward disease represents a unique pathogenic process governed by several polarized immune reactions and mechanisms. In this process, the deposition of respirable particulates stimulates dynamic pulmonary barrier immune and inflammatory responses. The initial responses aim to eliminate particles through type 1 inflammation but would convert to type 2 inflammation in the continued presence of particles in lung airways and parenchyma. Type 2 inflammation promotes the resolution of type 1 inflammation and repair of damaged tissue to maintain the pulmonary homeostasis through type 2 effector cells, such as M2 macrophages, and secreted mediators, such as SPMs, IL-10, and TGF-β. Under pathological conditions, prolonged and heightened type 2 inflammation takes place and promotes the development of interstitial fibrosis, granuloma formation, and tumorigenesis. Particle-stimulated fibroproliferative matrix alteration begins during the early phase inflammatory response, manifesting fibroblast proliferation, migration, and polarization into myofibroblasts, and increased production of collagens, which are boosted through M2s and TGF-β1 during type 2 inflammation.

Central to this time-dependent evolvement of type 1 and type 2 inflammation is the polarization of regulator and effector immune cells, including Th1 lymphocytes, ILC1s, and M1 macrophages during type 1 inflammation, and Th2 lymphocytes, ILC2s, and M2 macrophages during type 2 inflammation, stimulated by the persistent presence of inhaled particles and tissue injury. The progression of type 1 inflammation to type 2 inflammation and, ultimately, disease pathology is also regulated through several additional immune cell-mediated responses, which include the polarization of proinflammatory Th17 lymphocytes and immunosuppressive Tregs and Bregs, and expansion of MDSCs from perturbed myelogenesis. These polarized immune cells modulate the balance among T eff cells, i.e., Th1s, Th2s, and Th17s, thereby modulating the pathological processes relating to airway allergy, granuloma formation, interstitial matrix remodeling, tumor immunity, and immunotolerance. Some of these polarized cells, such as MDSCs, may also directly modulate downstream immune reactions and pathogenic alterations.

In the light of a large variation among micro and nano particulates in their size, shape, aspect ratio, rigidity, and other physicochemical properties, it is rational to posit that their interaction with the immune systems in the lung differ from one another substantially, which would affect their deposition and clearance in the lung and their inflammatory, fibrotic, tumorigenic, and autoimmune effects. Although much has been learned about the correlation between the adverse effects of inhaled particles and some of their properties, such as a large aspect ratio, high rigidity, and resistance to degradation, the knowledge on how these properties impact the interaction of different particles with immune cells remains sparse at the present time. On the other hand, there are clearly common and shared disease phenotypes among different particles, fibers, and nanomaterials, as discussed in more details earlier. Therefore, it is likely that pathogenic particulates with similar potencies and pathologic effects interact with the lung immune systems to induce the polarization of immune cells and responses similarly to each other. While evidence supporting this notion awaits further detailed investigation in near future, such information has translational implications for the safety evaluation and the safe design and utility of numerous micro and nano size particulate materials and products.

**Figure 3:**
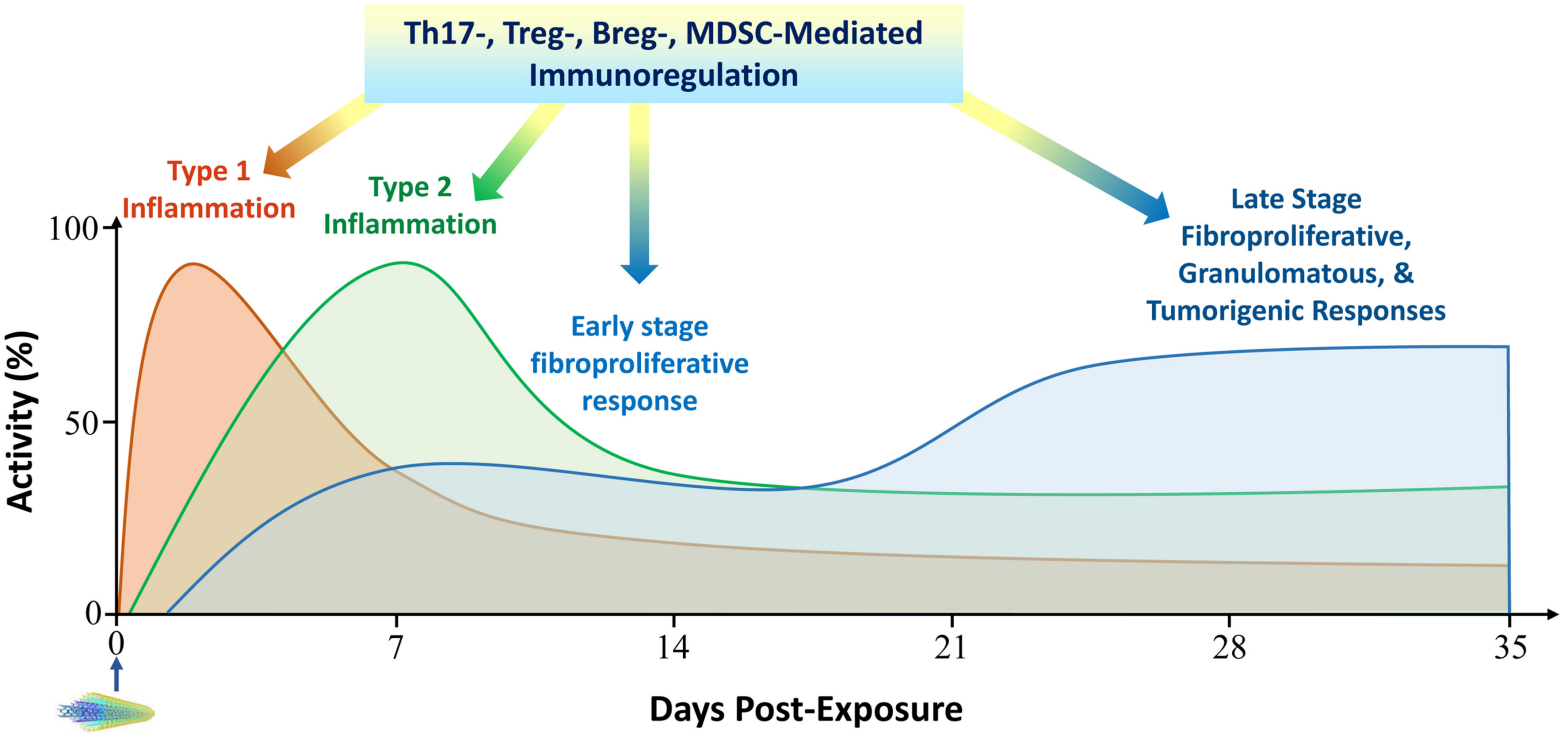
A working model to integrate pulmonary immune mechanisms in particle pathogenesis. The pulmonary response to inhaled particulates is largely governed by the interactions between particles and the pulmonary barrier immunity through several polarized immune responses. In this context, the time-dependent evolvement of type 1 to type 2 inflammation seemingly provides a foundation for the initiation and progression of the pathological development in the lung, which is characterized by continued local inflammation and cell death, fibrotic and granulomatous development, a pro-tumorigenic microenvironment, and propensity to autoimmune dysfunction. This dichotomous Th1 and Th2 progression is modulated by several other immune cell-mediated reactions toward pathologic development, which include the polarization of Th17s that amplifies proinflammatory reactions, the emergence of Tregs and Bregs that alters the balance among T eff cells toward disease-associated Th2 phenotypes, and the expansion of MDSCs from disturbed myelogenesis that infiltrate inflammatory and cancerous tissues and inhibit protective host immune reactions. Activity and time scales are for illustration purpose. Multi-walled carbon nanotubes (MWCNTs) are shown to represent particles administered to lungs at time zero. Breg, regulatory B cell; MDSC, myeloid-derived suppressor cell; Th, T helper; Treg, regulatory T cell.

These new findings and concepts suggest a working model for pulmonary disease pathogenesis elicited by inhaled particles and nanoparticles as summarized in Figure 3. Given the increasing, renewed interest in understanding the fundamental basis of particle-induced pulmonary disease and malignancy, and increased concerns on the safe production and utility of nanotechnology and nanoproducts, this working model can help generate new testable hypotheses to delineate the interaction between inhaled particles and the pulmonary immune system critical for the development of particle-induced disease outcomes at molecular and cellular levels in future studies.

## Conclusion

Inhaled particulates represent a unique class of sterile threats to the structure and function of the lung. Exposure to these micro- and nano-sized particles can lead to severe disease outcomes, including fibrosis, granulomatous inflammation, AHR, cancer, and autoimmune dysfunction. Recent evidence reveals that the deposition of inhaled particulates in the airways and alveoli sets forth a tissue response that is designed to eliminate the particles from the lung and to repair damaged tissue. This response is largely governed by the interactions between inhaled particles and the pulmonary barrier immunity through several polarized immune responses. In this framework, the time-dependent evolvement of type 1 to type 2 inflammation seemingly provides a foundation for the initiation and progression of the pathological development in the lung, which is characterized by continued local inflammation and cell death, formation of granulomas to enclose particle deposits, heightened production, remodeling, and hardening of the extracellular matrix, tumorigenesis in the lung and the pleura, and loss of immune self-tolerance. This dichotomous Th1 and Th2 paradigm is modulated by several other immune cell-mediated reactions during disease development. These include the polarization of Th17s that amplifies proinflammatory reactions, differentiation of Tregs and Bregs that alters the balance among T eff cells toward disease-associated Th2 phenotypes, and last but not the least, expansion of MDSCs that infiltrate inflammatory or tumorous tissues and boost the pathologic development by inhibiting host immune defense. These new findings and concepts on particle-induced polarization of immune cells and its role in particle pathogenesis reveal new mechanistic aspects of disease development induced by inhaled particles, which provides a framework for generating new hypothesis, identifying biomarkers, and designing new therapeutic strategies against particle-induced pathological effects and diseases.

## Author Contributions

QM is the sole contributor of this work and approves its publication.

## Conflict of Interest

The author declares that the research was conducted in the absence of any commercial or financial relationships that could be construed as a potential conflict of interest.

## Abbreviations

AHR: airway hyperreactivity
ALOX5AP: arachidonate 5-lipoxigenase-activating protein
ALOX15: arachidonate 15-lipoxigenase
AM: alveolar macrophage
AOP: adverse outcome pathway
ARG1: arginase 1
α-SMA: α-smooth muscle actin
BAL: bronchoalveolar lavage
Breg: regulatory B cell
C60F: fullerene C60
CNT: carbon nanotube
CTLA-4: cytotoxic T-lymphocyte-associated protein 4, CD152
DAMP: danger-associated molecular pattern
DC: dendritic cell
ENM: engineered nanomaterial
FIZZ1: found in inflammatory zone 1
FoxP3: forkhead box P3
GATA-3: GATA-binding protein 3
G-MDSC: granulocytic MDSC
HDM: house dust mite
HLD: hilar lymph node
IFN: interferon
Ig: immunoglobulin
IL: interleukin
ILC: innate lymphoid cell
iNOS: inducible nitric oxide synthase
i.n.i.: intranasal instillation
IPEX: immune dysregulation, polyendocrinopathy, enteropathy X-linked
IRF: interferon regulatory factor
i.t.i.: intratracheal instillation
LLC: Louise lung cancer
LPS: lipopolysaccharides
LTB4: leukotriene B4
M1: classically activated macrophage
M2: alternatively activated macrophage
MARCO: macrophage receptor with collagenous structure
MDSC: myeloid-derived suppressor cell
M-MDSC: monocytic MDSC
MHC: major histocompatibility complex
MMP: matrix metallopeptidase
MPF: massive progressive fibrosis
MSU: monosodium urate
MRC1: mannose receptor C-type 1, CD206
MWCNT: multi-walled carbon nanotube
NF-κB: nuclear factor kappa-light-chain-enhancer of activated B cells
NDMA: N-nitrosodimethylamine
NK: natural killer cell
NLRP3: NOD-like receptor family, pyrin domain containing 3
NO: nitric oxide
OVA: ovalbumin
p.a.: pharyngeal aspiration
PAMP: pathogen-associated molecular pattern
PD-1: programed cell death protein 1
p.i.: pharyngeal instillation
PMN: polymorphonuclear leukocyte
PRR: pattern recognition receptor
PSLT: poorly soluble particles of low toxicity
RELMα: resistin-like molecule α
ROR: retinoic acid receptor-related orphan receptor
ROS: reactive oxygen species
RNS: reactive nitrogen species
RvD: resolvin D
RvE: resolvin E
Smad: Sma and Mad related family
SPM: specialized pro-resolving mediator
ST2: IL-1 receptor-like 1
STAT: signal transducer and activator of transcription
SWCNT: single-walled carbon nanotube
TARC: thymus and activation regulated chemokine, CCL17
TAM: tumor-associated macrophage
T eff: CD4+ effector T cell, CD4+ T helper cell
T_FH_: follicular helper T cell
TGF: transforming growth factor
Th: T helper cell
TLR: Toll-like receptor
Treg: regulatory T cell
TSLP: thymic stromal lymphopoietin
w.b.i.: whole body inhalation
Yam1: chitinase 3-like 3.
